# The Power of Analogies for Imagining and Governing Emerging Technologies

**DOI:** 10.1007/s11569-018-0315-z

**Published:** 2018-05-15

**Authors:** Claudia Schwarz-Plaschg

**Affiliations:** University of Vienna, Universitätsstraße 7, 1010 Vienna, Austria

**Keywords:** Analogy, Imagination, Anticipation, Rhetoric, Emerging technologies, Nanotechnology, Responsible research and innovation (RRI)

## Abstract

The emergence of new technologies regularly involves comparisons with previous innovations. For instance, analogies with asbestos and genetically modified organisms have played a crucial role in the early societal debate about nanotechnology. This article explores the power of analogies in such debates and how they could be effectively and responsibly employed for imagining and governing emerging technologies in general and nanotechnology in particular. First, the concept of analogical imagination is developed to capture the explorative and anticipatory potential of analogies. Yet analogies do not simply stimulate imagination, they also restrict it by framing emerging technologies in specific ways. Thus, second, the article argues that tracing the rhetorical and persuasive power of analogical arguments is essential for understanding how analogies are constructed to legitimise assessments, funding policies, and governance approaches. Third, the article addresses factors that account for the persuasiveness of analogies in debates about emerging technologies. The article concludes with reflections on how analogical imagination and an enhanced analogical sensibility for framing and persuasive effects can foster responsible research and innovation (RRI).

## Introduction

When human beings encounter something new, they often seek to grasp its meaning and relevance by identifying similarities with better known phenomena. This so-called analogical reasoning is generally understood as a cognitive process in which knowledge from one domain, the source, is mapped onto a target [1]. The existence of such analogical processes also makes sense from an evolutionary perspective: if a berry resembles the berry that killed my fellow human being, I better not eat it or I might die too [2, 3]. While this represents a relatively simple example of how analogy by means of visual likeness is employed to ensure human survival, things get more complicated when it comes to highly complex, far-reaching, and—from a lay person’s perspective—invisible new technological and scientific developments such as nanotechnology.^1^ A definitive judgement about whether nanotechnology might nourish humanity and the planet or rather entail potential risks and unexpected implications is therefore much harder to attain. In the meantime, humanity has not only developed powerful technologies but also elaborated procedures and measurement instruments for scientific risk assessment that seek to estimate, anticipate, and weigh their benefits and risks. From the late 1980s onwards, such natural scientific assessments have been enriched by explorations of the ethical, legal and social aspects (ELSA) of new technologies, leading up to the current wave of activities under the rubric of responsible research and innovation (RRI). These scholarly movements seek to produce a different kind of anticipatory knowledge by stimulating scientific and non-scientific actors to reflect on the role of newly emerging science and technology in society. Hence, current approaches in RRI are less restricted to mapping and assessing impacts but more interested in fostering early collaboration and reflexivity among stakeholders in research and development processes [4]. While these processes have become more and more sophisticated, the involved actors—regardless of whether they are scientists, policy makers or “lay” citizens—are still very much analogical animals in the sense that they rely on analogies when thinking and debating about emerging technological developments.

An overview of the literature confirms the ongoing relevance of analogical thinking in science and technology contexts over the past decades. Bioethicists have explored the relevance and limits of specific analogies in debates on emerging bio- and nanotechnology [5–8]. Philosophers of science and science and technology studies (STS) scholars have carved out the innovation potential of analogies and metaphors^2^ in scientific knowledge production, where they contribute to conceptual change by making whole epistemic communities think of a phenomenon from a novel perspective [9–15]. Research on mental models has revealed that analogical reasoning is likewise an important heuristic for lay people making sense of technological systems and their underlying scientific concepts [16–18]. More recently, studies on public engagement with science and technology have highlighted that analogies are central devices for understanding and assessing emerging technologies in dialogue settings [19–25]. Media studies show that analogies and metaphors assist journalists in communicating complex scientific issues and concepts to a broader public [26–29]. Similarly, analogies represent essential tools in science education to convey abstract scientific concepts [30–32].^3^ This vast number of studies clearly demonstrates that analogical processes deserve and are also getting close attention in fields analysing the production, communication, and societal negotiation of technoscientific knowledge.

Yet this existing research suffers from two main shortcomings. First, the current body of work on stakeholder and public dialogue exercises hints at the relevance of analogies in these contexts, but it does not address explicitly how the power of analogies can be harnessed to explore and anticipate the development and societal implications of emerging technologies. To fill this gap, the first part of this article develops the concept of analogical imagination and thus points to an important but hitherto largely undervalued capacity among those who are involved in public and policy debates on emerging technologies. This section addresses primarily those researchers and actors who have come to play an active role in shaping technology governance as facilitators of stakeholder debates and public dialogue events in recent years. As will be argued, analogies can be important resources to guide and enrich such processes for a variety of reasons. Second, the majority of the literature mentioned above follows a cognitive paradigm that conceives of analogies as sense-making tools [35, 36] and thereby sidelines their argumentative and persuasive role in societal and academic debates about emerging technologies [37]. To balance this rather one-dimensional cognitive perspective, the article carves out an alternative rhetorical lens that allows to perceive the framing and persuading purposes of analogical arguments. This section hence adds an analytical dimension to the first, normative argument—it can also be seen as pointing to the other, darker side of analogical imagination: namely to the fact that imagination is always already imbued with specific interests and framings. The subsequent section, then, reflects on what makes analogies persuasive and in particular what I call the power of “the shared”. Although the basis and scope of this article is mainly conceptual, it also includes exemplary cases from the area of nanotechnology to demonstrate the empirical viability of the approach. Finally, I draw the main argumentative strands together and propose that developing analogical imagination and enhancing actors’ analogical sensibility for rhetorical effects can contribute to and strengthen the current normative commitment towards RRI in Europe and beyond.

## Analogical Imagination: Stimulating Exploration and Anticipation

In this first section, I seek to capture the power of analogies for stimulating deliberation on the social aspects and the anticipation of the future trajectories of emerging technologies with the concept of “analogical imagination”. Imagination is a complex, multifaceted concept with a long history in human thought [38]. For the purpose of this article, I define imagination with recourse to Paul Ricoeur as the power of the possible that can assist in teasing out the potentialities of reality [39, 40]. The aim of this section is to develop a conception of how harnessing analogical imagination can contribute to collective exploration and anticipation processes.

### Analogical Exploration: Accounting for Context, Process, and Innovation

The term analogical imagination first of all highlights that via the construction of analogies, the various characteristics and possible implications of a new technology can be explored. More specifically, analogical imagination helps to understand the relation of new and emerging technologies to other technologies or cases, because drawing analogies allows us to “come to know something about that object case over and above its existence as an allegedly isolated occurrence” (Smith 2002 [41]: 246). Thus, it assists in situating a new technology in a specific historical and sociotechnical context. Such contextualisation is not to be understood as a one-way mapping from one domain onto another, as argued by proponents of the mental models approach [1], but rather as a continuous, interactive making of relations between different cases. The conceptual interaction between cases should better be conceived of as symmetrical, which means that both the source and the target are co-created in the analogical process.^4^ From such a perspective, the main goal of analogical imagination lies not in gaining particular results but in the process itself because it stimulates imagination beyond the isolated case and helps to develop a contextual understanding. This conceptualisation has two benefits.

First, it accounts for the fact that drawing just one analogy does not suffice to grasp all relevant dimensions of new technoscientific fields. This is particularly true for nanotechnology, which represents not one homogenous technological field but rather acts as a placeholder for a broad range of technologies with potential applications in diverse areas. In contrast to frameworks such as the mental models approach, which consider inconsistencies between multiple models or analogies as problematic [35, 36], the framework of analogical imagination regards “inconsistencies” as expression of the complexity of the issue and its different dimensions [43]. Building multiple—also contradicting—analogies then does not signify inability but imaginative ability.

Second, analogical imagination regards analogies as situationally constructed for specific purposes and hence refrains from treating analogies as stable, immutable objects. This conception focuses on their flexible and temporary character, thus moving beyond an understanding of analogies as single, robust and completed entities, seeing them as necessarily multiple, unstable and incomplete. Against this background, rather than trying to establish one robust or perfect analogy, the creation of an ongoing dialogue about analogies as a means to identify a variety of relevant issues represents a more productive and realistic approach. This entails an understanding of analogical imagination not as something belonging to individual minds, but as being accomplished discursively in specific social settings, where analogies emerge out of collective thinking and negotiating processes.

This conception of analogical imagination is to some extent already actualised in engagement settings into which members of the general public and other societal stakeholders are invited to deliberate on nanotechnology and other emerging technologies. Exploration via analogical imagination takes place frequently in such dialogue-oriented contexts by itself [19–25, 37, 44], as participants naturally draw multiple analogies in order to explore the topic. To illustrate this point, Table 1 gives an overview of the central analogies that emerged in a public engagement setting on nanofood in the Austrian context [44, 45].^5^ This brief overview (in chronological order, lines in the transcript and speakers have been omitted for simplicity reasons) shows how a diverse set of analogies enabled the group to address a broad variety of issues ranging from potential risks and regulatory possibilities to the societal acceptance/rejection of nanofood. It also shows that the case of genetically modified (GM) food served as a prominent analogical source in the discussion of nanofood. But rather than encountering one GM-nano analogy, each time GM and nanotechnology are compared, a different analogy with a specific, contextual meaning emerges.

**Table 1:** Analogies in a public engagement setting on nanofood

Analogy with	Issue	Line of discussion and argumentation
Nuclear energy	Mistrust in expert opinions	Reference to the debate about nuclear energy in the 1970s and how experts made inconsistent predictions concerning the decomposition of radioactive material
GM food	Consumer sovereignty	Critique of how genetically modified (GM) foods were sold (“it is good for you”); argument that such promises would also not be acceptable with nanotechnology
Asbestos	Risk anticipation and regulation	Long-term consequences were unknown with asbestos and it could be similar with nanotechnology; demands for regulation
Medicine	Regulation	Nanotechnology should be as strictly regulated as medicine
GM food	Industry benefits	Comparison with GM food to argue that only producers will benefit from nanotechnology
Functional food	Societal acceptance	Not only discussion of potential consumer benefits of nanofood but also questioning of the promised benefits as “just marketing”; the analogy also suggested that nanofood might sell as well as existing functional food products
GM food	Societal acceptance and labelling	If nanofood were labelled like GM food, it would not be accepted by consumers
Mobile phones	Risk anticipation and societal acceptance	With certain new technologies (mobile phones), long-term risks are not properly studied; distinction between nanotechnological domains: in some (e.g. electronics), nanotechnology is more acceptable than in others (e.g. food)
X-rays	Hypes and risk anticipation	X-rays as an example for a new technology that was hyped and entailed collateral damage
GM food	Labelling and regulation	GM food labelling shows that regulation has flaws; if it were analogous with nanotechnology, regulation would be meaningless
GM food	Societal rejection	The GM food case illustrates the possibility of a similar societal rejection of nanofood

Third, analogical exploration in the above sense can provide innovative perspectives and not simply perpetuates or reproduces what is already known, as some suggest [47, 48]. To explicate the productive character of analogical imagination, I draw on Paul Ricoeur’s unpublished “Lectures on Imagination”, in which he distinguishes between reproductive and productive imagination [49]. Ricoeur argues that reproductive imagination relies on the mode of original and copy. Understanding analogies as transferring all features of a source onto a target in a copy-paste manner would count as reproduction, but this would arise from an overly narrow definition of analogical processes as being only about categorisation—the drawing of similarities. In reality, particularisation [50]—the drawing of distinctions or disanalogies—is always co-present in analogical processes as people hardly ever take the whole case but merely aspects of it as the basis for analogy-making (see Table 1). The problem thus disappears when differences remain acknowledged and the specificities of a new technology are not lost. Analogies hence rather resemble Ricoeur’s understanding of productive imagination. This type of imagination offers new descriptions of reality and finds its expression in utopian fiction as well as in epistemological and poetic imagination. Epistemological imagination produces new models of reality in science, and metaphor represents the main vehicle of productive imagination in poetry. Analogical imagination is a central element in both of these modes of productive imagination, because analogies are often at the core of new scientific paradigms [11] and metaphors are rooted in analogy [1]. Productive imagination does not come from nowhere; it is rooted in existing knowledge while simultaneously transforming it. In order to stay connected and meaningful to the present, imagination must “draw from existing reality sufficiently so that its productive distance is not too great” (Taylor 2006 [49]: 98). Thus, drawing on existing cases does not automatically lead to continuing what is known but rather is a prerequisite for combining knowledge in new ways. Like any form of meaning-generation, analogical imagination opens up moments for innovation/change or stabilisation/reproduction, depending on the elements that are brought together. I would further argue that an analogy can count as innovative or productive when it destabilises and transgresses entrenched ways of seeing and understanding (aspects of) the world. Working out new or less obvious similarities can thus draw attention to areas and issues that have been hitherto overlooked.

### Anticipation via Analogy: the Power of Retrospective Prospection

Imagination can be directed into different temporal directions: it connects an understanding of what is with what could be or has been, thereby always trying to move beyond the perceptual present, the here-and-the now. Thinking back to something in the past is always a way of imagining, just like it is to think of future possibilities. Recently, the notion of imagination has gained relevance in academic debates on emerging technologies mainly due to its prospective dimension, where it is thus often equated with anticipation. In this context, anticipation is considered to be a central capacity for envisioning potential safety, ethical, social or legal implications of relevance for the governance of emerging technologies [51]. As evident in Table 1, most analogies in this specific dialogue setting were anticipatory, that is, they did not establish how nanotechnology is but how it could potentially be or become like. With the turn to upstream engagement [52–54], facilitators of such dialogue processes have been confronted with the challenge as to how to stimulate anticipatory imagination at an early stage of development, when a technology is still in the making. One widespread approach to tackling this challenge has been to use scenarios, envisioned future applications and science or morality fiction to encourage anticipation among stakeholders and members of the public [55–62]. In the following, I put forward three reasons for why analogical imagination can work as a valuable counterbalancing force to such future-oriented tools in debates about nanotechnology and other emerging technologies.

First, facilitators of engagement processes should prevent discussions from drifting off into extreme futuristic scenarios and mere speculation [63], which often reproduces rather than critically scrutinises the promissory scientific and policy discourse accompanying emerging technologies. In order to avoid such tendencies, participants can be prompted to think back to how society has responded to previous technologies and the consequences these responses entailed. Here, analogies can unfold their retrospective-prospective power. In contrast to scenarios or science fiction, which can be developed without explicitly drawing on past experiences and cases, analogical imagination intrinsically incorporates such a retrospective dimension if the sources are stemming from actual occurrences (the exception would be analogies with fictional examples). In this sense, analogies work similarly to what Brown and Michael [64] call “prospecting retrospects”; a notion coined to describe how people use memories of once imagined sociotechnical futures—in particular with regard to their promises and (un)fulfilment—to construct new futures. By bringing this retrospective element to the fore, analogies can act as powerful resources in anticipatory processes and should be seen as complementary to more future-oriented devices such as scenarios or visions. Anticipatory analogies ground discussions about emerging technologies in a historical continuum, while still stimulating prospective imagination in upstream debates.

Second, it is through analogical imagination that actors can draw lessons from the past for the future [65–67], which is simply not possible by only looking at the future. Despite the fact that previous technologies are never tailor-made analogical sources for new technologies, they can still supply important learning experiences that can be used for enhancing anticipatory capacities, especially with regard to governance processes [7, 68, 69]. Again, this does not imply to do what has always been done and eschew from developing new regulatory frameworks but to stay open to past cases and experiences because meaningful decision-making needs to build on existing traditions, precedent cases and culturally established norms and values [70].

The third reason speaking for the retrospective-prospective potential of analogies relates to the debate about whether nanotechnology generates new ethical and social issues, and hence the existence of a separate field of “nanoethics” is justified [71, 72]. While it is certainly important to pay attention to the novel dimensions of new technologies, newness should not be the only or decisive aspect in debates and reflections on nanotechnology. If we solely focus on novelty, we run the risk of disregarding issues that are not new but nevertheless still relevant [73, 74]. Analogical imagination thus represents one way to counteract newness-centeredness, because it indicates which issues re-emerge or remain salient with nanotechnology and other emerging technologies. As Table 1 indicates, the issues that appear via analogies are drawn from past experiences but they are still considered relevant for members of the public, which is why they should likewise continue to play a role in political and academic debates.

## Analogical Arguments: Analysing the Power of Framing and Persuasion

After having made the case for the benefits of stimulating analogical imagination in societal debates about emerging technologies, I now turn to a more analytical perspective. What is missing in current frameworks is a clear conceptualisation of analogies’ double-edged character: namely that they not only are productive means of enhancing imagination but also represent a limiting force by restricting imagination. Assuming that debates about emerging technologies are merely about stimulating imagination would ignore that people have agendas that go beyond leading a rational or deliberative discussion. Imagination is never neutral but includes a specific framing of the world [37]. For this reason, analogical arguments represent the other side of analogical imagination. This section aims to foreground that when analogies are mobilised in debates, they are employed as powerful argumentative devices to construct persuasive versions of the world, while pushing aside other possible versions. This is generally not made explicit and reflected upon in contemporary analyses of the role of analogies in science and technology contexts—especially when social scientists and humanists have to fulfil dual roles as facilitators and analysts. By stimulating deliberation in stakeholder and public engagement, the imaginative dimension of analogies (see above) ultimately becomes overemphasised. The rhetorical lens presented in the following allows to bring the neglected framing and persuasive dimension back into view.

Following Potter (1996 [75]: 33), I understand “rhetoric as discourse used to bolster particular versions of the world and to protect them from criticism”. This definition highlights that rhetorical discourse creates versions of reality—it is constitutive—and it stresses the strategic role of rhetoric in defending one version over others—it is argumentative and hence aims to persuade. Seen through this lens, analogies create specific understandings of reality by establishing similarities and simultaneously shielding them against potential counter-arguments. This perspective is closely connected to studies on framing effects. The term “framing” is generally employed to refer to how a social situation is understood [76], and experiences are turned into meaningful narratives [77–80]. Research on framing acknowledges the key role of analogies and metaphors, among other devices, in the construction of frames [81–83]. Similarly, analogies can be understood as central resources in the construction, stabilisation or undermining of sociotechnical imaginaries [69, 84, 85].

From the above follows that anticipatory analogies [37] are not mere prospective tools but likewise work to present certain future scenarios as more plausible than others. Scholarship in the Sociology of Expectations tradition has highlighted the performative role of expectations and promises in research and development processes, especially the effect of future-oriented rhetoric on the present [64, 86]. Analogies are crucial resources for underpinning expectations and promises, as different social groups make use of different analogies to mobilise for their preferred version of the future. Since analogies primarily actualise their persuasive power in the early stages of a political debate [87] or technological development, when people have not yet developed an opinion and pathways can still be shaped, it is especially relevant to pay attention to their framing character during this period. Once established and accepted, analogies have already ordered knowledge, formed our conception and anticipation of reality and influenced the way people position themselves and act towards new technologies [88].

Analogies hence need to be approached as strategic devices. Struggles or agreement over analogies can inform us about what is considered to be a relevant or disputed frame of comparison, in other words, what should count as a relevant type of similarity, which in turn brings more latent values, concerns and interests to the fore. In arguing about their analogies, people argue about much more than merely what counts as a valid comparison. Changing the frame of comparison is an effective way to shift attention away from one set of values to another. Controversies about analogies thus represent an ideal entry point for analysing underlying values, visions and social dynamics in societal debates about emerging technologies.

### Tracing Analogical Arguments: Examples from Nanotechnology

In the following, I apply the rhetorical lens to four cases from the field of nanotechnology to exemplify how it can be used to trace and interpret the power of analogical arguments. The cases are presented in chronological order of their appearance in the debate about nanotechnology, thus also allowing to identify a shift in the overall discourse and the role of specific analogies in it.

1. In the early 2000s, nanotechnology emerged as a new and promising field of research and development in the technopolitical arenas of Western nation states. During this period, scientists and political actors drew various analogies between nanotechnology and previous culturally influential technological developments to highlight its potential impact and mobilise for nanospecific funding initiatives. For example, the foundational report for the National Nanotechnology Initiative of the USA anticipated via analogy that “the effects of nanotechnology on the health, wealth, and lives of people could be at least as significant as the combined influences of microelectronics, medical imaging, computer-aided engineering, and man-made polymers developed in this century” (National Science and Technology Council et al. 2000 [89]: 13). Although this passage starts out like a mere vision (“could”), the way it expects nanotechnology to excel the combined impact of four preceding technological developments conveys an implicit certainty about nanotechnology’s even higher potential. A similar phrasing can be found in a policy document from the Austrian Council for Research and Technology Development, which was published 2 years after the US report and sought to promote a nanospecific funding programme, the Austrian Nano Initiative. It imagined and framed nanotechnology as a “promising future technology with an enormous application potential in many industrial sectors and areas of life [which] could have a similarly strong impact on our civilization as did information and communication technologies over the last decade” (Rat für Forschung und Technologieentwicklung 2002 [90]: 1). In contrast to the US imagination, the Austrian expectation appears more modest, because it just compares nanotechnology’s potential to that of information and communication technologies. Nonetheless, both examples demonstrate that parallels with other influential technologies are drawn to generate excitement for nanotechnology, legitimise funding initiatives and hence are clearly invoked for persuasive purposes. Such analogical arguments underpinning promissory technoscientific futures can often be encountered in the early stages of debates about emerging technologies, and they clearly indicate that a specific version of reality is promoted.
2. The asbestos-nano analogy [91] surfaced a few years after the initial promissory analogies in the scientific, policy and media discourse on nanotechnology. By foregrounding material similarities between carbon nanotubes and asbestos fibres, the analogy suggests that nanoparticles could turn out equally harmful in the future. Although the asbestos-nano analogy did not build on existing scientific evidence, the mere argument for possibility again provided a sufficient argumentative basis for the examination of the health and environmental effects of nanoparticles [73]. It can thus be not only interpreted as a prevention-oriented pointer for regulators to learn from past mistakes, but it also allowed to channel the debate about nanotechnology in terms of safety, health and environmental risks that can be examined by natural sciences such as (eco-)toxicology. References to past cases such as asbestos and CFC, where at first promissory materials turned out problematic in the long turn, were employed by central scientific figures to argue (successfully) for more risk research on nanoparticles and the funding of (eco-)toxicological research [92]. At the same time, however, the prominence of asbestos and other risk-foregrounding analogies in political and public debates on nanotechnology also elucidates a narrowing of the debate on risk rather than innovation governance [93]. Whereas the former frames nanotechnology merely in terms of its safety, health and environmental impacts,^6^ the latter is also interested in questions about its ethical and societal dimensions as well as the broader sociotechnical visions and innovation models it enacts.
3. The genetically modified organism (GMO)-nano analogy appeared almost simultaneously with the asbestos analogy around 2003, when scholars and policy makers began to consider nanotechnology against the backdrop of public controversies over GMOs in Europe [7, 8, 95].^7^ On the European policy level, the public backlash against green biotechnology was framed as a political failure that should not be repeated with nanotechnology. The need to learn from the GM debate in order to avoid a similar public controversy was hence palpable in conferences and policy papers [96, 97] as well as in pamphlets by social scientists [98, 99]: “the GM experience represents a warning, a cautionary tale of how not to allay public concern. Avoiding nanotechnology becoming ‘the next GM’ is seen as critical to the public acceptability of applications in the field” (Kearnes et al. 2006 [99]: 15). The GMO-nano analogy was hence employed to encourage and legitimise attempts to respond to potentially negative public reactions in a proactive manner. It entailed specific policy actions and played a key role in establishing public engagement and communication initiatives as well as social scientific research into nanotechnology’s ethical and social dimensions, especially in the USA and Europe [7, 100, 101]. Although the GMO-nano analogy also comes up frequently in public engagement settings among lay participants [19, 20, 37, 69], the way it is constructed there (see also Table 1) runs counter to its use in policy contexts. The GMO analogy is commonly established in public engagement when people discuss applications (such as nanofood) that seem to hold hardly any benefits for consumers or citizens [102]. Lay people tend to frame the GMO opposition movement in a positive way, that is, it is drawn upon as a successful template for how civil society can influence technological trajectories and resist specific innovations against powerful political and industrial interests [69]. Rather than being spaces where public acceptance is produced, public engagement settings are thereby re-framed as spaces where existing power relations and top-down governance approaches are challenged. This is achieved, among other means, by re-imagining analogies that already exist in the policy arena and thus creating an alternative framing.
4. The last case illustrates the rhetorical use of analogies with material from a podium discussion at a “Young Researcher Symposium” in Vienna, Austria. This 1-day event was held in 2012 and marked the closing of a project that engaged students around the age of 17 years in reflecting on the risks and benefits of nanotechnology. The podium discussion was composed of several students, who had participated in the project, and stakeholder representatives, among them a politician from the Austrian Ministry of Life, a spokesperson from a consumer protection organisation and an industry representative from the Austrian Chamber of Commerce. Excerpt 1 below reveals how the different stakeholders used analogies right at the beginning of debate to achieve specific persuasive effects and foster their agendas.

### Excerpt 1^8^

1. Moderator: To what extent is the fascination with nano comparable to the fascination with radioactivity back in the day?
2. Politician: That’s an interesting analogy because last year we had Marie Curie year. But you can’t compare it with nano, because nano’s portfolio is much larger and the societal assessment much better. Its applications are already quite concrete, so that we can grasp the fascination better.
3. Consumer representative: The comparison is not quite fitting. Radioactivity has a clear effect, nano has a lot of different effects, a much larger spectrum, which however is also the problem with nano. Another comparison would be with asbestos because the fibres are similar. It was already observed around 1900 that asbestos causes lung disease, but it was not banned by law until 1990. We need to do research on the negative effects of nano to guarantee its responsible introduction.
4. A female student: I agree with the consumer representative. Back then with radioactivity everything was new and what do they do now with nano? It’s also a new technology that is not fully researched and we already use it! The comparison can be made. We have to be careful. I would be really careful.
5. Industry representative: This analogy is misleading. We are now much more advanced with risk anticipation. In the area of food, nano has been used since the 1960s. Nano is nothing new, we have been using it for long now. Of course you have to do risk assessment, but to just say that a product with nano is dangerous would be wrong.
6. A male student: You can compare it because the fascination is similar. Radioactivity was also en vogue back then. It’s true, it has been used since the 19th century, but there’s still work to do until you can say it’s okay.
7. Another male student: If you think about thalidomide—and with nano the examination is still missing. We will hit on it 20 years later.
8. Industry representative: Yes and no. A lot of products pass through long processes, for instance medicine and cosmetics. There’s precise risk assessment, it’s not true that industry doesn’t take responsibility.
9. Politician: Thalidomide is a great example because we really learned a lot since then. We’ve tried to improve the licensing systems and to handle our non-knowledge. Also with CFC we were so happy with its properties. All we can do is to make our systems better. We can’t guarantee that something like thalidomide won’t happen again.

In turn 1, the moderator puts an analogy with radioactivity up for debate, which suggests a similar societal “fascination” attending both the introduction of radioactivity and nanotechnology. The debate immediately takes off as different viewpoints are articulated with regard to the analogy, leading also to the construction of other analogies as the adequacy of the radioactivity analogy is challenged and defended. The students and the consumer representative use analogies with radioactivity, asbestos and thalidomide to highlight the potential health threats nanotechnology could pose and to demand better risk assessment. Although they do not address the industry representative and politician directly, the fact that these two stakeholder representatives react with defence strategies clearly indicates that they feel responsible for the safe development of nanotechnology. In turn 5, the industry representative tries to appease risk concerns by arguing that nanotechnology is not fundamentally new.^9^ The politician in turn 9, like the industry representative in turn 8, seeks to regain public trust by highlighting that state regulators have learned from past failures in risk assessment. However, he also stresses that politicians cannot be blamed if nanotechnology turns out problematic in the long term, because there exists no absolute certainty that “something like thalidomide” will never occur again. The debate in Excerpt 1 nicely shows that analogies invoking shared technohistorical knowledge can provide fruitful starting points for stimulating debate about a new technology, but likewise that analogies are used rhetorically because stakeholders construct and judge analogies from their specific standpoint. They formulate their analogical arguments to present nanotechnology in a specific light, to communicate their hopes and concerns and to attribute and fend off responsibility.^10^

All four cases demonstrate that a framing-sensitive, rhetorical perspective allows to capture that analogies are never simply drawn to learn from the past for mere imaginative reasons [67] but that they are constructed for specific purposes and are used to legitimate funding policies, engagement initiatives and other communication activities, as well as governance and regulatory approaches [47, 105]. Attending to the argumentative and persuasive features of analogies hence inescapably brings power structures and struggles over authority into view. Attempts to introduce or change the dominant analogies are tied to actor interests and their specific vision of what nanotechnology might entail for society.

## The Power Behind Persuasive Analogies

The first three case studies above represent examples of analogies that involved observable real-world consequences such as the establishing of multimillion dollar and euro funding programs, academic institutes, projects dedicated to risk and social scientific research and public engagement exercises. A relevant question is thus what makes analogies persuasive (i.e. being accepted as being “true”) and hence represents a prerequisite for making them performative (i.e. having perceivable, material effects).

Analogies do not meet the standards of absolute proof, that is, there exists no logical rationale why two phenomena must be the same, work in the same manner or have the same consequences just because they share some characteristics [106]. What makes analogies apt for rhetorical investigation is thus precisely that their persuasiveness does neither rely on logical validity nor upon empirical or scientific evidence [107]. Although some may hold on to the idea that the value of an analogy can be judged by criteria such as whether the compared aspects are of relevance, dissimilarities annihilate the similarities, and parallels are sufficient to support the conclusion [108]; these criteria do not sufficiently explain how and why some analogies manage to become accepted, while others fail to accomplish this effect. One step towards an explanation is to acknowledge that social, political and cultural factors contribute to their persuasiveness. Some analogies might simply be persuasive due to the credibility and societal status of their enunciators. When people engage with a new technology, these engagements are always “socially mediated in the sense that judgments of trust will have to be made regarding whose analogies, metaphors, and so on are credible” (Michael and Brown 2004 [23]: 381). The persuasive power of an analogy hence also depends on the power and trustworthiness of the people who push the analogy. Consider the promissory analogies in the policy documents above, where analogies are constructed by already powerful actors to legitimise political actions.

A second key factor contributing to analogies’ persuasiveness is what I call the power of “the shared”. Analogies enact this power by evoking widely shared interpretations in their audiences. Rhetorical accounts of metaphor and analogy emphasise that speakers and listeners need to share a “system of associated commonplaces” [42] to guarantee successful communication. The term “commonplace” also alludes to the necessity of commonplace-specific knowledge underlying effective analogies, because a commonplace is literally a shared geographical space. Thus, persuasive analogies are often built from a common knowledge source with which both speakers and audience are familiar. As the four cases from the nanofield above demonstrate, analogical sources tend to represent technologies and materials whose impacts are known widely, sometimes even globally. Events with a global media coverage hence represent unique source material for the construction of analogical arguments or broader sociotechnical imaginaries [85]. However, at the same time, national context also matters for how these sources are interpreted. While the risks of technologies such as nuclear energy may transgress national borders and supranational organisations may hold a certain power in shaping governance frameworks, the societal debate and regulation of emerging technologies is still to a large extent a national governance issue. For instance, in public engagement settings where people of heterogeneous backgrounds meet, who might not share more than residence in the same geographical area, knowledge of a shared national technopolitical history is particularly relevant. Participants in such engagement settings frequently draw on nation-specific knowledge by means of analogy to imagine, convey and defend their visions of new technologies [20, 69, 84, 109, 110]. Regardless of whether events are global, national or local in scope, in order to function as persuasive analogical sources, they need to be part of the collective cultural repertoire of a group and ideally entail a widely shared meaning.

By conjuring up such shared interpretations of previous experiences, analogies can influence how people attach meaning to a new scientific or technological field. Thus, analogies frame by evoking shared interpretations or framings. A transference of framings will be particularly persuasive when a frame is attached to a master frame or an archetypical story that is based on long-standing cultural interpretations and narrations about how societies deal with emerging technologies [69, 111]. Analytically, a focus on analogies can thus serve as an avenue to examine “culturally specific, historically and politically grounded, public knowledge-ways” or “civic epistemologies” (Jasanoff 2005 [79]: 254), which form the basis for what counts as trustworthy argument or evidence in a specific cultural or national context. Such a perspective assists in understanding divergences in governance approaches and public opinions with regard to emerging technologies and the role that analogies play in stabilising existing framings. In contrast to other resources such as expert knowledge, personal experience or science fiction [20], which may not be shared by the majority in a national context, analogical references to cases taken from technopolitical history are based on a more widely shared cultural knowledge base and hence will generate a more persuasive force.

## Discussion and Conclusion

The aim of this article was twofold. First, it sought to lay out a novel perspective for utilising the imaginative power of analogies in societal debates about emerging technologies, especially in those contexts that currently go by the name of public and stakeholder engagement. To highlight the explorative and anticipatory potential of analogical processes, the concept of *analogical imagination* was developed. A commitment to analogical imagination demands that we do not succumb to the idea that one single analogy can or should guide debates or policy-making [8, 105], but that the power of analogies lies in generating open-ended, explorative discourse. Analogical imagination in this sense is akin to a mode of deliberation, which is characterised by a swinging back and forth between different analogies. In engagement contexts, this would mean to encourage participants to construct multiple analogies and test them out in an experimental manner. This flexible stance stands in contrast to the determined advocate who has already decided upon an analogy or argument to be endorsed and defended (cp. Billig 1987 [50]: 156). Thus, using analogical imagination may be one possible avenue to avoid or break up polarised positions in societal debates about emerging technologies. To use analogical imagination as such, a productive force means to engage in carving out dissimilarities, constructing alternative analogies and in creating an awareness for what analogies do. This also implies to eschew simplistic assumptions of analogies as merely being sense-making and imaginative tools.

To gain an understanding of what analogies (are meant to) do, the second aim of the article was to present a rhetorical lens through which framing and persuasive effects become visible. This lens should not be seen as optional but necessarily complementary to gain a more complete picture of what analogies are used for. Coming back to the berry example from the beginning, there is something at stake when people draw analogies. The fact that analogies are powerful devices in constructing views of reality and legitimising actions calls for a finely tuned *analogical sensibility*. I conceptualise analogical sensibility as the outcome of detailed analyses and reflexive practices that excavate implicit framings and attempts to persuade. By tracing analogies and counter-analogies from a rhetorical perspective, as was exemplified with the four nanocases, the argumentative topography that co-emerges in debates around emerging technologies can become visible and provide the basis for a deeper reflection of the different forces that seek to steer their development in a given society.

Both the practical fostering of analogical imagination in debates and the development of analogical sensibility via analysis and reflection can contribute to efforts towards *responsible research and innovation* (RRI) [112]. By opening up research and innovation processes to society and diverse stakeholders, RRI aims to make research and innovation activities more “transparent, interactive process by which societal actors and innovators become mutually responsive to each other with a view on the (ethical) acceptability, sustainability and societal desirability of the innovation process and its marketable products” (Von Schomberg [113]: 9). While RRI can be framed rather instrumentally as the application of several strategies (as does the European Commission with its focus on public engagement, science education and literacy, open access, gender, ethics, governance), it can also be understood as an attempt to foster more general capacities such as *anticipation, inclusion, reflexivity* and *responsiveness* in governance debates about emerging technologies [114]. What I propose is that analogical imagination and analogical sensibility can enhance these four, often entangled capacities and thus also strengthen actors’ orientation towards RRI in significant ways. In the following, I will therefore discuss how analogies may assist in cultivating these capacities and also come up with more practical suggestions for how this could be achieved.

First, as argued in the “Anticipation via Analogy: the Power of Retrospective Prospection” section, analogical imagination can historically ground anticipation processes due to its intrinsic retro-prospective character. Hence, analogical imagination can provide the footing without which anticipation remains a merely speculative, non-contextual undertaking. It also allows actors to learn from past mistakes for the future. At the same time, all involved actors need to be careful not to assume that analogical imagination can be capable of providing clear solutions, templates or guidelines for how to act with regard to highly uncertain emerging technologies. Much rather analogical imagination should be understood as one integral part in the cumulative production of anticipatory knowledge. Second, to unfold its full potential, analogical imagination has to be inclusive, which means that it has to include a broad variety of societal and stakeholder perspectives via engagement and dialogue exercises. Public engagement settings are important spaces in which counter-analogies to the more promissory analogies mobilised by policy and industry actors can emerge [37]. Facilitators and social scientists then are responsible to bring these alternative analogies and framings into the broader public and policy discourse. If policy makers really seek to implement the inclusiveness that RRI and engagement activities promise, public opinions need to be integrated into governance systems, even if they run counter to dominant narratives of technoscientific progress. Third, analogical sensibility is a necessary ingredient for enabling reflexivity because it allows to see the framing, persuading and performative effects of analogies as well as the ways in which they contribute to myth building and stabilisation [115]. An understanding of these effects is a prerequisite to strengthen the reflexive capacity of those actors who shape the future of emerging technologies. As Stilgoe et al. (2013 [114]: 1571) argue, institutional reflexivity “means holding a mirror up to one’s own activities, commitments and assumptions, being aware of the limits of knowledge and being mindful that a particular framing of an issue may not be universally held”. In this vein, developing analogical sensibility should make actors reflect on the analogies that are “so deeply embedded in the language and traditions of a community of interpretation that the user is not directly aware of them” (Post and Leisey 1995 [108]: 47). Policy makers, industry and scientific actors who use analogies deliberately to foster their (research) agendas should commit to the responsible use of analogies, which implies the willingness to put one’s own analogies up for critical scrutiny. Likewise, citizens participating in public engagement activities should be supported in thinking through existing analogies, in order to gain reflexivity about their constructed nature and power in shaping discourse. This may consequently enable them to come up with alternative analogies, thus practising analogical imagination and developing analogical sensibility can become part of a broader effort towards democratisation in science and technology governance. Fourth and finally, the dimension of responsiveness points to the “capacity to change shape or direction in response to stakeholder and public values and changing circumstances” (Stilgoe et al. 2013 [114]: 1571). With regard to analogies, responsiveness means to stay open for the new knowledge and positions that co-emerge in an ongoing process of using collective analogical imagination. Responsiveness also requires social scientists to explore how the societal debate about emerging technologies such as nanotechnology changes via the mobilisation of specific analogies and to respond adequately in circumstances where certain analogies may come to dominate by proposing other alternative analogies.

In the year 2018, analogies appear to be no longer centre stage in the political and academic debate about nanotechnology—which may indicate, if nothing else, that nanotechnology has become an established rather than emerging technology. At the same time, many issues with regard to nanotechnology’s governance are far from resolved. In order to continue a productive societal discussion about nanotechnology, it can therefore be helpful to use stakeholders’ analogical imagination to further explore and anticipate the social and ethical challenges of more specific nanotechnological applications. Perhaps it is time for some new analogies to be born? Following the analogical approach proposed in this article could be one conceivable way through which the conversation could be spurred again.

## Footnotes

1 I use the term nanotechnology here very broadly for all research and development activities that study and/or manipulate matter on the nanoscale (1 to 100 nm).

2 Analogy and metaphor are closely related because both are based on a comparative process. Hence, they are often treated synonymously in everyday speech and even academic contexts. But it is also important to see their differences: metaphors are figures of speech, whereas analogies refer more generally to the ways in which phenomena are presented to be alike to deduce their likeness in another dimension.

3 Studies on the press coverage of nanotechnology [33] and nanoengineering education [34] have observed that analogies to human hairs, ants, or houses are mainly used to convey the nanoscale dimension.

4 For more details on such an interactional view in the context of metaphor, see [42]

5 This discussion group was one out of four such public engagement events that were conducted in the project “Making Futures Present: On the Co-production of Nano and Society in the Austrian Context” (P 20819, PI: Ulrike Felt) at the Department of Science and Technology Studies, University of Vienna. For more on these discussion groups and their methodology, see Felt et al. [45, 46].

6 As a review of the epistemological underpinnings and status quo of scientific risk assessment of nanomaterials shows, a variety of major obstacles render the risk-focused approach infeasible for supporting decision-making and regulation on nanotechnology [94].

7 For a detailed exploration of the parallels between the debate about the social and ethical dimensions of nanotechnology and GM food, see Sandler [7]

8 This excerpt has been translated from the original German version by the author and proofread by an English native speaker.

9 This represents a well-known rhetorical move among nanotechnology stakeholders [103].

10 This corresponds with an analysis of public meetings among nanotechnology stakeholders in the Netherlands, in which stakeholders from industry and civil society organisations participated primarily to send their messages and to play their attributed roles instead of engaging in a more open two-way dialogue [104]. A more practical question that arises from such findings is how facilitators may enable collective imagination and open up solidified analogical framings.

## References

[1] Gentner D, Bowdle BF, Wolff P, Boronat C, Gentner D, Holyoak KJ, Kokinov BK (2001). Metaphor is like analogy. The analogical mind: perspectives from cognitive science.

[2] Mervis CB, Rosch E (1981). Categorization of natural objects. Ann Review of Psych.

[3] Bar M (2007). The proactive brain: using analogies and associations to generate predictions. Trends Cogn Sci.

[4] Oftedal G (2014). The role of philosophy of science in responsible research and innovation (RRI): the case of nanomedicine. Life Sci Soc Policy.

[5] Hofmann B, Solbakk JH, Holm S (2006). Teaching old dogs new tricks: the role of analogies in bioethical analysis and argumentation concerning new technologies. Theor Med Bioeth.

[6] López JJ (2006). Mapping metaphors and analogies. Am J Bioeth.

[7] Sandler R, Gordijn B, Cutter AM (2014). GM food and nanotechnology. In pursuit of nanoethics.

[8] Sandler R, Kay WD (2006). The GMO-nanotech (dis)analogy?. Bull Sci Technol Soc.

[9] Pitt JC (2011). Doing philosophy of technology: essays in a pragmatist spirit.

[10] Knorr-Cetina K (1981). The manufacture of knowledge: an essay on the constructivist and contextual nature of science.

[11] Hesse MB (1966). Models and analogies in science.

[12] Leatherdale WH (1974). The role of analogy, model, and metaphor in science.

[13] Maasen S, Weingart P (2000). Metaphors and the dynamics of knowledge.

[14] Montuschi E, Radman Z (1995). What is wrong with talking of metaphors in science?. From a metaphorical point of view: a multidisciplinary approach to cognitive content of metaphor.

[15] Hallyn F (2000). Metaphor and analogy in the sciences.

[16] McCloskey M (1983). Naive theories of motion.

[17] Gentner D, Gentner DR, Gentner D, Stevens AL (1983). Flowing waters or teeming crowds: mental models of electricity. Mental models.

[18] Kempton W, Holland D, Quinn N (1987). Two theories of home heat control. Cultural models in language and thought.

[19] Burri RV (2009). Coping with uncertainty: assessing nanotechnologies in a citizen panel in Switzerland. Public Underst Sci.

[20] Davies SR (2011). How we talk when we talk about nano: the future in laypeople’s talk. Futures.

[21] Horlick-Jones T, Walls J, Kitzinger J (2007). Bricolage in action: learning about, making sense of, and discussing issues about genetically modified crops and food. Health Risk Soc.

[22] Marková I, Linell P, Grossen M, Orvig Salazar A (2007). Dialogue in focus groups: exploring socially shared knowledge.

[23] Michael M, Brown N (2004). The meat of the matter: grasping and judging xenotransplantation. Public Underst Sci.

[24] Wibeck V, Abrandt Dahlgren M, Öberg G (2007). Learning in focus groups: an analytic dimension for enhancing focus group research. Qual Res.

[25] Marcu A, Gaspar R, Rutsaert P, Seibt B, Fletcher D, Verbeke W, Barnett J (2015). Analogies, metaphors, and wondering about the future: lay sense-making around synthetic meat. Public Underst Sci.

[26] Anderson AG, Petersen A, Wilkinson C, Allan S (2009). Nanotechnology, risk and communication.

[27] Hellsten I, Nerlich B, Bucchi M, Trench B (2008). Genetics and genomics: the politics and ethics of metaphorical framing. Handbook of public communication of science and technology.

[28] Väliverronen E (2004). Stories of the “medicine cow”: representations of future promises in media discourse. Public Underst Sci.

[29] Gschmeidler B, Seiringer A (2012). “Knight in shining armour” or “Frankenstein’s creation”? The coverage of synthetic biology in German-language media. Public Underst Sci.

[30] Aubusson P, Harrison AG, Ritchie S (2006). Metaphor and analogy in science education.

[31] Wormeli R (2009). Metaphors & analogies: power tools for teaching any subject.

[32] Filliettaz L, de Saint-Georges I, Duc B (2010). Skiing, cheese fondue and Swiss watches: analogical discourse in vocational training interactions. Vocat Learn.

[33] Ten Eyck TA, Hernandez P (2009). Metaphor usage in early press coverage of nanotechnology: turning science into soccer balls and human hair. Open Soc Sci J.

[34] York E (2015). Smaller is better? Learning an ethos and worldview in nanoengineering education. NanoEthics.

[35] Bostrom A (2008). Lead is like mercury: risk comparisons, analogies and mental models. J Risk Res.

[36] Collins A, Gentner D, Holland D, Quinn N (1987). How people construct mental models. Cultural models in language and thought.

[37] Schwarz-Plaschg C (2018). Nanotechnology is like…The rhetorical roles of analogies in public engagement. Public Underst Sci.

[38] Stevenson L (2003). Twelve conceptions of imagination. Br J Aesthet.

[39] Ricoeur P (1965). History and truth.

[40] Ricoeur P (1978). The metaphorical process as cognition, imagination, and feeling. Crit Inq.

[41] Smith B (2002). Analogy in moral deliberation: the role of imagination and theory in ethics. J Med Ethics.

[42] Black M (1962). Models and metaphors: studies in language and philosophy.

[43] Wynne B, Jasanoff S, Markle GE, Petersen JC, Pinch T (1995). Public understanding of science. Handbook of science and technology studies.

[44] Schwarz CG (2014) Nano is like…The role of analogies in public engagement with nanotechnology in Austria. Doctoral thesis at the University of Vienna, Department of Science and Technology Studies

[45] Felt U, Schumann S, Schwarz CG, Strassnig M (2014). Technology of imagination: a card-based public engagement method for debating emerging technologies. Qual Res.

[46] Felt U, Schumann S, Schwarz-Plaschg C (2018) IMAGINE—a card-based discussion method. In: Liamputtong P (ed) Handbook of research methods in health social science. Springer, Singapore, forthcoming

[47] Johnson S, Burger I (1996). Limitations and justifications for analogical reasoning. Am J Bioeth.

[48] Lessnoff M (1997). The role and limits of analogical argument: a reply to Aronovitch. Polit Stud XLV.

[49] Taylor GH (2006). Ricoeur’s philosophy of imagination. J Fr Francoph Philos.

[50] Billig M (1987). Arguing and thinking: a rhetorical approach to social psychology.

[51] Barben D, Fisher E, Selin C, Guston DH, Hackett EJ, Amsterdamska O, Lynch M, Wajcman J (2007). Anticipatory governance of nanotechnology: foresight, engagement, and integration. The handbook of science and technology studies.

[52] Joly P-B, Kaufmann A (2008). Lost in translation? The need for “upstream engagement” with nanotechnology on trial. Sci Cult.

[53] Wilsdon J, Willis R (2004). See-through science: why public engagement needs to move upstream.

[54] Macnaghten P (2008). Nanotechnology, risk and upstream public engagement. Geography.

[55] Rip A, te Kulve H, Fisher E, Selin C, Wetmore JM (2008). Constructive technology assessment and socio-technical scenarios. The yearbook of nanotechnology in society 1: presenting futures.

[56] Swierstra T, Stemerding D, Boenink M, Sollie P, Düwell M (2009). Exploring techno-moral change: the case of the ObesityPill. Evaluating new technologies: methodological problems for the ethical assessment of technology development.

[57] Türk V, Knowles H, Walbaum H, Kastenholz H (2005) Nanologue scenarios: the future of nanotechnology. We need to talk. Report of the Nanologue project

[58] Bennett I, Fisher E, Selin C, Wetmore JM (2008). Developing plausible nano-enabled products. The yearbook of nanotechnology in society 1: presenting futures.

[59] Goorden L, Van Oudheusden M, Evers J, Deblonde M, Fisher E, Selin C, Wetmore J (2008). Nanotechnologies for tomorrow’s society: a case for reflective action research in Flanders, Belgium. The yearbook of nanotechnology in society 1: presenting futures.

[60] Andersen I-E, Jæger B (2001). Scenario workshops and urban planning in Denmark. Particip Learn Action.

[61] Selin C (2011). Negotiating plausibility: intervening in the future of nanotechnology. Sci Eng Ethics.

[62] Mensvoort K, van Wouters S, Vos C, Konrad K, Coenen C, Dijkstra A (2013). NANO supermarket: using speculative design to catalyze a technology debate. Shaping emerging technologies: governance, innovation, discourse.

[63] Nordmann A (2007) If and then: a critique of speculative nanoethics. NanoEthics 1:31–46

[64] Brown N, Michael M (2003). A sociology of expectations: retrospecting prospects and prospecting retrospects. Technol Anal Strateg Manag.

[65] European Environment Agency (2002) Late lessons from early warnings: the precautionary principle 1896–2000. Office for Official Publications of the European Communities, Luxembourg

[66] European Environment Agency (2013) Late lessons from early warnings: science, precaution, innovation. Eea Report

[67] Von Schomberg R (2010) Introduction. In: Von Schomberg R, Davies S (eds) Understanding public debate on nanotechnologies: options for framing public policy. European Commission, Directorate-General for Research, Brussels, pp 5–12

[68] Kuzma J, Priest S (2010). Nanotechnology, risk, and oversight: learning lessons from related emerging technologies. Risk Anal.

[69] Felt U, Jasanoff S, Kim S-H (2015). Keeping technologies out: sociotechnical imaginaries and the formation of Austrian technopolitical identity. Dreamscapes of modernity: sociotechnical imaginaries and the fabrication of power.

[70] Aronovitch H (2007). The political importance of analogical argument. Polit Stud.

[71] McGinn RE (2010). What’s different, ethically, about nanotechnology? Foundational questions and answers. NanoEthics.

[72] Allhoff F (2007). On the autonomy and justification of nanoethics. NanoEthics.

[73] Van de Poel I (2008). How should we do nanoethics? A network approach for discerning ethical issues in nanotechnology. NanoEthics.

[74] Royal Commission on Environmental Pollution (2004). Nanoscience and nanotechnologies: opportunities and uncertainties.

[75] Potter J (1996). Representing reality: discourse, rhetoric and social construction.

[76] Goffman E (1974). Frame analysis: an essay on the organization of experience.

[77] Gamson WA, Modigliani A (1989). Media discourse and public opinion on nuclear power: a constructionist approach. Am J Sociol.

[78] Scheufele DA (1999). Framing as a theory of media effects. Aust J Commun.

[79] Jasanoff S (2005). Designs on nature: science and democracy in Europe and the United States.

[80] Snow DA, Benford RD (1988). Ideology, frame resonance, and participant mobilization. Int Soc Mov Res.

[81] Kuypers JA, Kuypers JA (2009). Framing analysis. Rhetorical criticism: perspectives in action.

[82] Entman RM (1991). Framing U.S. coverage of international news: contrasts in narratives of the KAL and Iran air incidents. Aust J Commun.

[83] Kitzinger J (2000). Media templates: patterns of association and the (re)construction of meaning over time. Media Cult Soc.

[84] Jasanoff S, Kim S-H (2015) (eds) Dreamscapes of modernity: sociotechnical imaginaries and the fabrication of power. University of Chicago Press, Chicago

[85] Hurlbut BJ, Jasanoff S, Kim S-H (2015). Remembering the future: science, law, and the legacy of Asilomar. Dreamscapes of modernity: sociotechnical imaginaries and the fabrication of power.

[86] Brown N, Rappert B, Webster A (2000). Contested futures: a sociology of prospective techno-science.

[87] Lynch J (2009) Does analogical reasoning affect political attitudes? Evidence from survey experiments. Doctoral thesis at Harvard University

[88] Wyatt S (2004). Danger! Metaphors at work in economics, geophysiology, and the Internet. Sci Technol Hum Values.

[89] National Science and Technology Council, Committee on Technology, Subcommittee on Nanoscale Science, Engineering and technology (2000) National Nanotechnology Initiative: The initiative and its implementation plan

[90] Rat für Forschung und Technologieentwicklung (RFT) (2002). Nanowissenschaften und -technologien: Gesamtkonzept. Empfehlung vom 14./15.2.2002.

[91] Kane AB, Hurt RH (2008). The asbestos analogy revisited. Nat Nanotechnol.

[92] Kelty C (2009). Beyond implications and applications: the story of ‘safety by design. NanoEthics.

[93] Felt U, Wynne B (2007) Taking European knowledge society seriously: report of the expert group on science and governance to the science, economy and society directorate, directorate-general for research. European Commission

[94] Miller G, Wickson F (2015). Risk analysis of nanomaterials: exposing nanotechnology’s naked emperor. Rev Policy Res.

[95] Burri RV, Jasanoff S, Sang-Hyung K (2015). Imaginaries of science and society: framing nanotechnology governance in Germany and the United States. Dreamscapes of modernity: sociotechnical imaginaries and the fabrication of power.

[96] European Commission (2007) Public communication and applied ethics of nanotechnology: learning from the GM debate. Second International Advanced Course, NanoBio-RAISE, 23 – 28 September 2007, St Edmund Hall, Oxford

[97] ANAP (2010) Austrian nanotechnology action plan. Federal Ministry of Agriculture, Forestry, Environment and Water Management, http://www.nanoinitiative.at/1560_EN.pdf, Wien

[98] Einsiedel EF, Goldenberg L (2004). Dwarfing the social? Nanotechnology lessons from the biotechnology front. Bull Sci Technol Soc.

[99] Kearnes M, Macnaghten P, Wilsdon J (2006). Governing at the nanoscale: people, policies and emerging technologies.

[100] Delgado A, Kjølberg KL, Wickson F (2011). Public engagement coming of age: from theory to practice in STS encounters with nanotechnology. Public Underst Sci.

[101] Bowman DM, Hodge GA (2007). Nanotechnology and public interest dialogue: some international observations. Bull Sci Technol Soc.

[102] Bosso C, Coenen C, Dijkstra A, Fautz C (2014). Nano risk governance, soft law, and the US regulatory regime. Innovation and responsibility: engaging with new and emerging technologies.

[103] Swierstra T, Rip A (2007). Nano-ethics as NEST-ethics: patterns of moral argumentation about new and emerging science and technology. NanoEthics.

[104] Krabbenborg L, Mulder HAJ (2015). Upstream public engagement in nanotechnology: constraints and opportunities. Sci Commun.

[105] McCray PW (2008) It’s just like that, except different: the power of analogy in describing nanotechnology. Sci Prog:92–94

[106] Mill JS (1879). System of logic: ratiocinative and inductive.

[107] McKinlay A, McVittie C (2008). Social psychology and discourse.

[108] Post SG, Leisey RG (1995). Analogy, evaluation, and moral disagreement. J Value Inq.

[109] Horst M, Irwin A (2010). Nations at ease with radical knowledge: on consensus, consensusing and false consensusness. Soc Stud Sci.

[110] Macnaghten P, Guivant JS (2011). Converging citizens? Nanotechnology and the political imaginary of public engagement in Brazil and the United Kingdom. Public Underst Sci.

[111] Macnaghten P, Davies SR (2010). Narratives of mastery and resistance: lay ethics of nanotechnology. NanoEthics.

[112] Owen R, Bessant J, Heintz M (2013). Responsible innovation: managing the responsible emergence of science and innovation in society.

[113] Von Schomberg R (2011) Introduction. In: Towards responsible research and innovation in the information and communication technologies and security technologies fields. European Commission, EC Directorate General for Research and Innovation, Luxembourg

[114] Stilgoe J, Owen R, Macnaghten P (2013). Developing a framework for responsible innovation. Res Policy.

[115] Torgersen H, Fuchs D (2017). Technology assessment as a myth buster: deconstructing myths around emerging technologies. J Responsible Innov.

